# *Clostridium perfringens* Delta-Toxin Induces Rapid Cell Necrosis

**DOI:** 10.1371/journal.pone.0147957

**Published:** 2016-01-25

**Authors:** Soshi Seike, Kazuaki Miyamoto, Keiko Kobayashi, Masaya Takehara, Masahiro Nagahama

**Affiliations:** Department of Microbiology, Faculty of Pharmaceutical Sciences, Tokushima Bunri University, Yamashiro-cho, Tokushima 770–8514, Japan; Wake Forest School of Medicine, UNITED STATES

## Abstract

*Clostridium perfringens* delta-toxin is a β-pore-forming toxin and a putative pathogenic agent of *C*. *perfringens* types B and C. However, the mechanism of cytotoxicity of delta-toxin remains unclear. Here, we investigated the mechanisms of cell death induced by delta-toxin in five cell lines (A549, A431, MDCK, Vero, and Caco-2). All cell lines were susceptible to delta-toxin. The toxin caused rapid ATP depletion and swelling of the cells. Delta-toxin bound and formed oligomers predominantly in plasma membrane lipid rafts. Destruction of the lipid rafts with methyl β-cyclodextrin inhibited delta-toxin-induced cytotoxicity and ATP depletion. Delta-toxin caused the release of carboxyfluorescein from sphingomyelin-cholesterol liposomes and formed oligomers; toxin binding to the liposomes declined with decreasing cholesterol content in the liposomes. Flow cytometric assays with annexin V and propidium iodide revealed that delta-toxin treatment induced an elevation in the population of annexin V-negative and propidium iodide-positive cells. Delta-toxin did not cause the fragmentation of DNA or caspase-3 activation. Furthermore, delta-toxin caused damage to mitochondrial membrane permeability and cytochrome *c* release. In the present study, we demonstrate that delta-toxin produces cytotoxic activity through necrosis.

## Introduction

Isolates of *Clostridium perfringens* type B and C are responsible for fatal diseases ranging from necrotizing enterocolitis to enterotoxemia in humans and livestock [1–3]. Delta-toxin is a basic protein (32-kDa) produced by certain strains of *C*. *perfringens* types B and C [1], but it remains unclear whether delta-toxin is a key pathogenic agent in these types. Delta-toxin induces hemolysis of sheep, goat, and pig erythrocytes, but the erythrocytes of the other species are inherently resistant [4–6]. Furthermore, the toxin disrupts various eukaryotic cells containing human monocytic cells, rabbit macrophages and platelets from rabbits, humans and goats [6–8], and it is also known to possess lethal activity [6, 9]. On the basis of these findings, delta-toxin has been considered to play an essential role in the virulence of type B and C strains.

Delta-toxin belongs to the *Staphylococcus aureus* alpha-toxin family of β-pore-forming toxins (β-PFTs) [9, 10]. Delta-toxin shows significant homology (about 40% identity) with *C*. *perfringens* beta-toxin, the contributing factor of Pig-bel in humans and necrotic enterocolitis in domestic animals, and to *C*. *perfringens* NetB, the cause of avian necrotic enteritis [9]. All three toxins are produced as monomers, which recognize membrane receptors on the target cell surface, and assemble into oligomers [10, 11]. The entire structure of delta-toxin is remarkably correlated with those of NetB and alpha-toxin [12]. Delta-toxin has a three-domain structure consisting of mainly β-sheets. A feature of the *S*. *aureus* alpha-toxin family of β-PFTs is the core stem domain of monomers containing three short β-strands packed against the β-sandwich [10, 12, 13]. Like alpha-toxin, delta-toxin and NetB are also arranged in three domains, β-sandwich, rim, and stem domains [10, 12, 14]. On the other hand, the rim domains of delta-toxin and NetB show sequence and conformational differences compared with alpha-toxin [10, 12, 14]. Because the rim domain of *S*. *aureus* alpha-toxin is important for binding to cell membrane receptors, the differences in these rim domains explain why delta-toxin and NetB bind to distinct receptors. In fact, alpha-toxin recognizes a protein receptor, whereas delta-toxin interacts with ganglioside GM2 [9, 11]. The receptor of NetB is still unclear.

The selective cytotoxic activity of delta-toxin is related to the recognition of GM2 ganglioside [4–6], and the toxin exhibits cytotoxicity only to cells expressing GM2 on their membranes. On the other hand, it has been reported that the toxin also binds to another membrane component [9,12]. However, the mechanism of delta-toxin-induced cytotoxicity is not fully understood. In this study, we investigated the cytotoxicity of delta-toxin in various cell lines and the functions of its oligomers using an artificial membrane. We found that delta-toxin killed five cell lines (A549, A431, Caco-2, Vero and MDCK), with A549 cells being most sensitive to the toxin. Therefore, to investigate the cytotoxic mechanism of delta-toxin, A549 cells provide a good model system. Here, we have analyzed cytotoxicity caused by delta-toxin using A549 cells and examined the actions of the toxin on mitochondria, which involve various types of cell death. These results show that delta-toxin causes cell necrosis in the target cells.

## Materials and Methods

### Materials

Methyl-β-cyclodextrin (MβCD), protease inhibitor mixture (100X), Z-VAD-FMK, protease inhibitor mixture, staurosporine, thrombin, 5(6)-carboxyfluorescein diacetate (CF), and sphingomyelin (SM) from bovine brain were obtained from Sigma-Aldrich (St. Louis, MO). Cholesterol and phosphatidylcholine (PC) from egg yolk were obtained from Nacalai Tesque (Kyoto, Japan). Antibodies against caveolin-1, flotillin, and β-actin were obtained from Santa Cruz Biotechnology (Dallas, TX). Cy3 Mon-Reactive Dye Pack, horseradish peroxidase-labeled goat anti-rabbit immunoglobulin G, horseradish peroxidase-labeled sheep anti-mouse immunoglobulin G, and an ECL Western blotting kit were obtained from GE Healthcare (Tokyo, Japan). Mouse anti-cytochrome *c* (6H2.B4) antibody was obtained from BD Bioscience (Tokyo, Japan). Hanks' balanced salt solution (HBSS), Alexa Fluor 488 cholera toxin subunit B, Alexa Fluor 568-conjugated goat anti-rabbit immunoglobulin G, MitoTracker Red CMXRos, Alexa Fluor 568-conjugated goat anti-mouse immunoglobulin G, Cellular Lights^TM^ Mito-GFP BacMam 1.0, Hoechst 33342, and Dulbecco's modified Eagle's medium (DMEM) were provided by Life Technologies (Tokyo, Japan). Antibodies against Bax (N-terminus) and Bak (N-terminus) were obtained from Merk-Millipore (Tokyo, Japan). Antibodies against active caspase-3 antibody and VDAC1 were purchased from Cell Signaling (Tokyo, Japan). Hanks' balanced salt solution (HBSS) and Dulbecco's minimal essential medium (DMEM) were purchased from Gibco BRL (New York, NY). Molecular weight markers for DNA electrophoresis were obtained from Takara Bio Inc. (Otsu, Japan). All other reagents were of analytical grade.

### Expression and purification of delta-toxin

A delta-toxin gene cloned into a pET28a vector was provided by Dr M. Popoff (Institute Pasteur, Paris, France). Recombinant delta-toxin was expressed as a 6×His tagged protein in *Escherichia coli* BL21 [9]. Briefly, after growth (at 26°C) and induction (1 mM isopropyl β-D-1-thiogalactopyranoside, Sigma-Aldrich), the bacteria were harvested, suspended in lysis buffer (50 mM Tris-HCl buffer, pH 8, 300 mM NaCl, and protease inhibitor mixture) and sonicated on ice. The cell lysates were separated from the soluble fraction by centrifugation (17,000 g, 20 min). The soluble fraction was applied to a cobalt affinity column (TALON, Takara-Bio, Tokyo, Japan). Thereafter, purification of recombinant toxin was performed in accordance with the manufacturer's instructions. Cleavage by thrombin was performed using a standard method [9].

### Preparation of anti-delta-toxin antisera

Antisera for delta-toxin were obtained from immunizing rabbits preinjected purified delta-toxin, as described previously [15]. Freund’s adjuvant complete (Becton, Dickinson and Company, Tokyo, Japan) was mixed with delta-toxin (50 μg) (1:1). Two intramuscular injections were given. Antiserum were prepared 2 weeks after the final injection.Western analysis revealed that anti-delta-toxin antiserum recognized purified delta-toxin but not beta-toxin produced by *C*. *perfringens* and reacted with a single 28kDa band, consistent with the known size of delta-toxin, in cell lysates of *E*. *coli* transformed with a recombinant delta-toxin plasmid. Experimental protocols were approved by the Institute Animal Care and Use Committee at Tokushima Bunri University.

### Cell culture

Human lung adenocarcinoma cells (A549), human epidermoid carcinoma cells (A431), human epithelial colorectal adenocarcinoma cells (Caco-2), African green monkey kidney cells (Vero), and Madin-Darby canine kidney epithelial cells (MDCK) were obtained from Riken Cell Bank (Tsukuba, Japan). Cells were cultivated in DMEM containing 10% fetal calf serum (FCS), streptomycin (100 μg/ml), penicillin (100 U/ml), and glutamine (2 mM) (FCS-DMEM). All culture steps were performed at 37°C in 5% CO_2_.

### Cell viability

The viability of cells was measured by the reduction of 3 -(4,5-dimethylthiazol-2-yl)-5-(3-carboxymethoxyphenyl)-2-(4-sulfophenyl)-2H-tetrazolium (MTS) (MTS assay; Promega, Tokyo, Japan). The absorbance was measured at 490 nm. The viability of cells was calculated as follows: mean absorbance of the toxin group/mean absorbance of the control [16].

### Lactate dehydrogenase release assay

Lactate dehydrogenase (LDH) activity in A549 cell culture supernatants was determined using an enzymatic assay (Wako Pure Chem., Osaka, Japan, LDH-Cytotoxic Test) in accordance with the manufacturer’s instructions. Positive control cell lysates were prepared with 0.2% Triton X-100 (= 100% cell death).

### Assay for cellular ATP

Cellular ATP contents were determined using a luminescence assay (Cell-Titer Glo Luminescent Cell assay; Promega) in accordance with the manufacturer's protocols. Cells were treated with various concentrations of delta-toxin at 4°C or 37°C for the indicated periods. The intensity of luminescence was determined using a TopCount NXT microplate scintillation luminescence counter (Perkin-Elmer, Tokyo, Japan) [17].

### Western blotting

Methods of sodium dodecyl sulfate-10% polyacrylamide gel electrophoresis (SDS-PAGE) and standard Western blotting, including the use of antibodies against delta-toxin, caveolin-1, flotillin, active caspase-3, cytochrome *c*, actin and VDAC1 were as described previously [16, 17].

### Liposomes

SM- and PC-cholesterol liposome-encapsulated CF were prepared as described previously [16, 18]. Release of CF was determined using a method reported previously [16, 18]. The binding to liposomes of delta-toxin was carried out using a method described previously [19].

### Isolation of lipid rafts

A549 cells grown on 35-mm-diameter dishes were treated with delta-toxin at 4°C for 30 min. Then, the cells were washed and incubated at 37°C for 1 h. All the following steps were carried out at 4°C. Cells were rinsed with HBSS, treated with 1% Triton X-100 for 30 min in HBSS containing a protease inhibitor mixture, and then sonicated with 20-s pulses (two times) using a tip-type sonicator. The lysates were adjusted to 40% sucrose (w/v), overlaid with 2.4 ml of 36% sucrose and 1.2 ml of 5% sucrose in HBSS, centrifuged at 45,000 rpm (250,000 g) for 18 h at 4°C in an SW55 rotor (Beckman Instruments, Palo Alto, CA), and fractionated from the top (0.4 ml each, a total of 10 fractions) [16]. The aliquots of the gradient fractions were subjected to SDS-PAGE and immunoblotting analysis. The cholesterol content of each fraction was determeined using a diagnostic kit (Cholesterol E-Test Wako; Wako Pure Chemical, Osaka, Japan) [16].

### Flow cytometry

Apoptotic and necrotic cell rates were evaluated by flow cytometry (FCM) analysis utilizing an Annexin-V-FLUOS staining kit (Roche, Tokyo, Japan). A549 cells were inoculated at 1 × 10^5^ cells per well in 24-well plates overnight. The cells were incubated with heat-inactivated delta-toxin (1 μg/ml) or delta-toxin (10 and 20 ng/ml) for indicated time periods at 37°C. Then, cells were washed with phosphate-buffered saline (PBS) (-) and detached using trypsin-EDTA. The recovered cells were carefully transferred to a 1.5 ml centrifuge tube, washed with ice-cold PBS (-) and centrifuged for 5 min at 1,100 g to remove the supernatant. Staining of the sample was carried out in accordance with the manufacturer's protocols, and more than 40,000 cells were scanned using a Guava easyCyte Flow Cytometer (Millipore, Tokyo, Japan). We carried out an analysis of quadrant population using FlowJo. The population of live cell was negative for both annexin V and propidium iodide (PI) (lower left quadrant).

### DNA fragmentation assay

A549 cells cultured in 35-mm-diameter tissue culture plates were treated in the absence or presence of delta-toxin (20 ng/ml) at 37°C for various time periods. After incubation, DNA was isolated using a Genomic DNA Purification Kit (Promega) in accordance with the manufacturer's protocols, and DNA (2 μg) was evaluated by electrophoresis in a 1.5% agarose gel, followed by staining with ethidium bromide [17].

### Measurement of cytocheome c release

The mitochondria fraction and cytosol fraction from A431 cella was isolated by different centrifugation. The cells were collected and washed 2 times with PBS, then were resupended in a sucrose containing buffer (1 mM EDTA, 250 mM sucrose, 25 mM Tris-HCl pH7.5 containing a protease inhibitor mixture) at 4°C for 60 min. The cells were homogenized and centrifuged at 1100 g for 15 min at 4°C to separate nuclei and cell debris. The fraction of mitochondia was then sedimented by centrifugation at 11,000 g for 30 min at 4°C. The supernatant fuid was centrifuged at 110,000 g for 50 min at 4°C to obtain the cytosol fraction.

### Immunofluorescence studies

Delta-toxin was labeled using Cy3 in accordance with the manufactuer's protocols (GE Healthcare). Cells were cultured on a polylysine-treated glass plate (MatTek Corp., Ashland, MA) at 37°C under 5% CO_2_ for 1 day in FCS-DMEM. To examine the binding of delta-toxin, A549 cells were treated with Cy3-labeled delta-toxin (1 μg/ml) for 30 min at 4°C. After four washes with cold FCS-DMEM, the cells were incubated with FCS-DMEM at 37°C for 15 min. The cells were washed three times with PBS (-) and treated with 3% paraformaldehyde fixative at room temperature, washed extensively with PBS (-), and viewed using a confocal microscope (Nikon A1 laser scanning confocal microscope, Nikon Corp. Tokyo, Japan) [17]. Nuclei of cells were visualized by Hoechst 33342 (20 μg/ml) staining. To examine the alteration of Bak and Bax and the liberation of cytochrome *c*, A549 cells were cultured at 37°C for 1 day on glass-bottomed plates. Cells were transfected with Mitochondria-GFP (Life Technologies) in accordance with the manufacturer’s protocols. After 24 h, the transfected cells were incubated with delta-toxin, fixed, permeabilized and blocked using the above-mentioned methods. Cells were stained with anti-Bax (N-terminus), anti-Bak (N-terminus), or anti-cytochrome *c* antibodies. Bound antibodies were detected using Alexa Fluor 568-labeled secondary antibodies. To investigate changes in mitochondria, A549 cells were treated with Hoechst 33342 and Mito-Tracker Red CMXRos for 30 min at 37°C [17]. Images were taken using a confocal microscope and processed with Adobe Photoshop.

### Statistical analysis

One-way analysis of variance (ANOVA), followed by Bonferroni’s multiple comparison test, was used to assess the means. Results are indicated as the mean ± standard deviation (SD). SD values indicate the variation among mean values obtained from at least four independent experiments. A *P* value of 0.05 or less was used for assessing statistical significance.

## Results

### Cytotoxicity of delta-toxin

**Fig 1:**
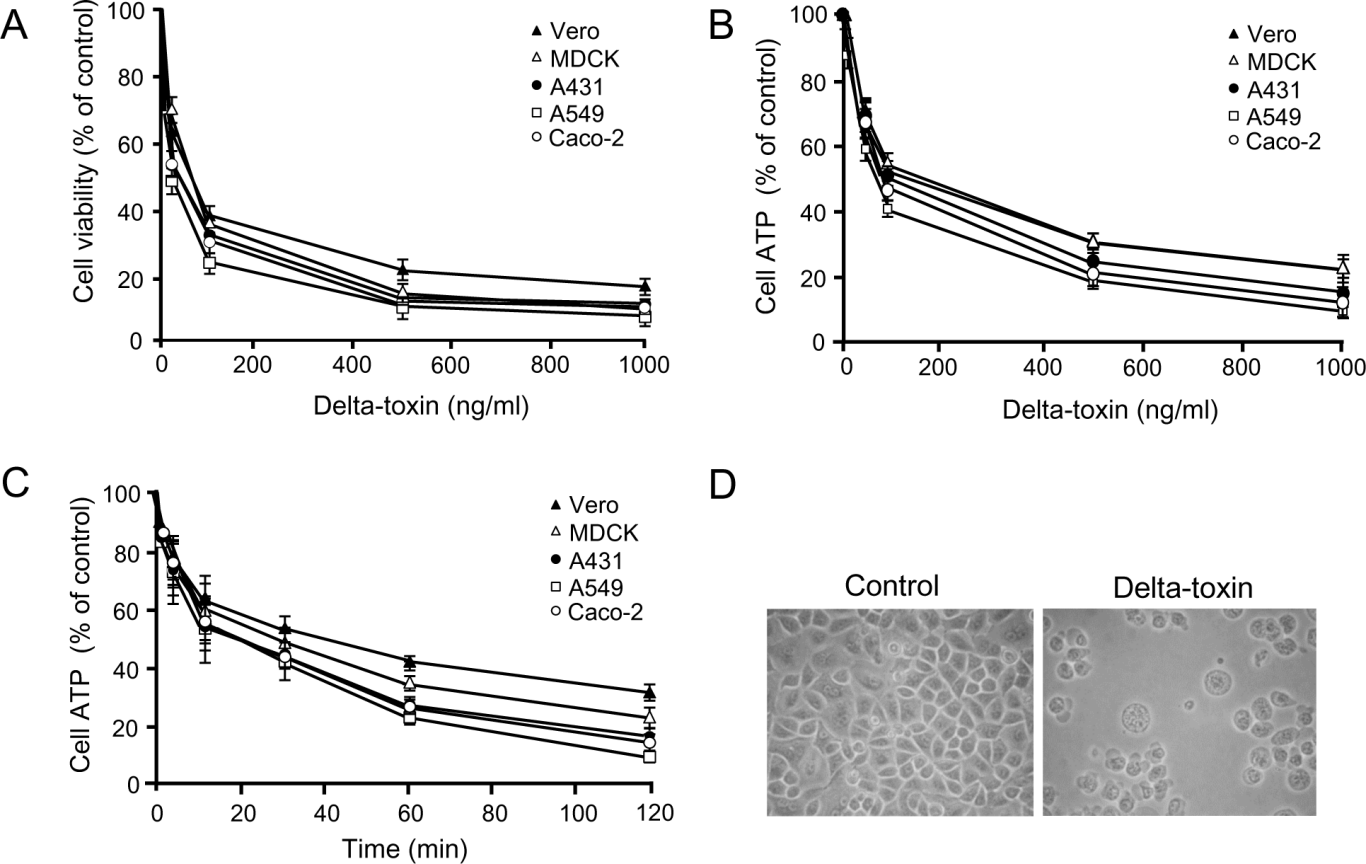
Cytotoxicity of delta-toxin. (A) Cells were incubated with delta-toxin for 1 h at 37°C. Viability of cells was assessed using the 3-(4,5-dimethylthiazol-2-yl)-5-(3-carboxymethoxyphenyl)- 2-(4-sulfophenyl)-2H-tetrazilum (MTS) method. (B) Cells were treated with delta-toxin for 1 h at 37°C prior to the ATP assay. (C) Cells were treated with delta-toxin (50 ng/ml) for the indicated time periods at 37°C prior to an ATP was assayed. Results are indicated as percentage of the value for controls. The mean ± standard deviation (SD) for four experimental studies is shown. (D) Cytotoxic effect of delta-toxin on A549 cells. A549 cells were incubated for 1 h at 37°C in the presence or absence of delta-toxin (50 ng/ml). Typical data from one of four experimental studies are shown. The cells were observed using phase-contrast microscopy. Magnification, ×150.

To investigate the cytotoxicity of delta-toxin, we used MTS assays with various cell types. Delta-toxin induced the death of A549, A431, Caco-2, Vero, and MDCK cells at 37°C (Fig 1A). Delta-toxin (above 100 ng/ml) decreased cell viability to less than 50% in all cell lines. Anti-delta-toxin antiserum neutralized the cytotoxicity of delta-toxin. On the other hand, heat-inactivated delta-toxin showed no cytotoxicity to these cells. Next, cell survival rate was evaluated by ATP measurements (Fig 1B and 1C). Delta-toxin decreased ATP contents in a dose- and time-dependent manner in all cell lines. To investigate the toxin-induced morphological changes of cells, A549 cells were exposed to the toxin (50 ng/ml) for 1 h at 37°C. In Fig 1D, cells displayed swelling and blebbing. The same damage was observed in other cells treated with delta-toxin. These findings indicated that delta-toxin shows cytotoxicity to various cells.

### Binding of delta-toxin to A549 cells

**Fig 2:**
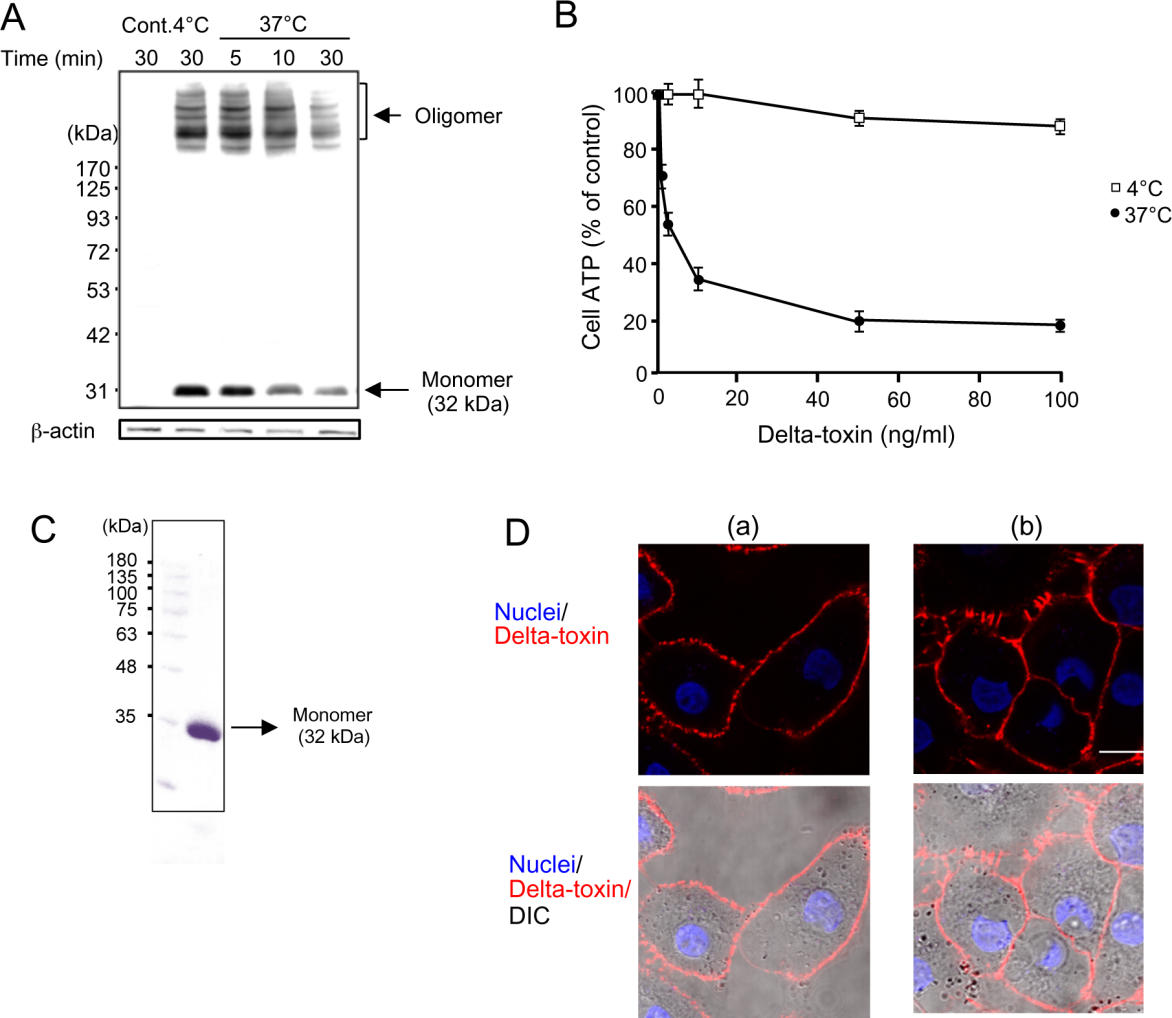
Binding and oligomerization of delta-toxin in A549 cells. (A) A549 cells were treated with delta-toxin (1 μg/ml) for 30 min at 4°C. The cells were washed, and incubated for the indicated time periods at 37°C. The cells were dissolved in SDS-sample solution without heating and confirmed by immunoblotting of delta-toxin and β-actin (control). Representative data from one of four experimental studies are shown. (B) A549 cells were treated with delta-toxin for 1 h at 4°C or 37°C prior to an ATP assay. Results are indicated as percentage of the value for controls. The mean ± standard deviation (SD) for four experimental studies is shown. (C) SDS-PAGE analysis of purified delta-toxin. The purified preparation of delta-toxin (5 μg of protein) from cell lysates of *E*. *coli* transformants containing a delta-toxin encoding plasmid was resolved using sodium dodecyl sulfate-polyacrylamide gel electrophoresis, followed by staining with Coomassie brilliant blue. Lanes: 1, molecular mass standards; 2, purified delta-toxin. (D) A549 cells were treated with Cy3-labeled delta-toxin (1 μg/ml) for 30 min at 4°C (panel a). After washing, cells were cultivated with medium for 30 min at 37°C (panel b). The cells were fixed using formaldehyde and stained with Hoechst 33342. Cy3-labeled delta-toxin (red) and the cell nuclei (blue) were examined using a confocal microscope. DIC, differential interference contrast. The images are representative of those from four experimental studies. Bar, 5 μm.

Delta-toxin forms an oligomer with itself on the plasma membrane of HeLa cells [9]. To examine the binding and oligomer formation of delta-toxin on A549 cells, the cells were incubated with delta-toxin for 30 min at 4°C. Then, the cells were washed and incubated at 37°C for the indicated time periods (Fig 2A). Cells were lysed and subjected to immunoblotting analysis using antiserum against delta-toxin (Fig 2A). When the A549 cells were treated with delta-toxin at 4°C, monomer and oligomer of delta-toxin were detected. Upon transfer of cells from 4°C to 37°C, the levels of delta-toxin monomer did not change but the bands decreased in intensity after 30 min (Fig 2A). Heat-inactivated delta-toxin did not bind to the cells. On the other hand, analysis of delta-toxin by SDS-PAGE showed the presence of the monomer of the toxin in solution (Fig 2C). To study the relationship between the formation of oligomer and the cytotoxicity of delta-toxin, we compared the cytotoxicity at 4°C and 37°C using intracellular ATP measurements (Fig 2B). A549 cells were treated with various concentrations of delta-toxin at 4°C or 37°C for 1 h. Although delta-toxin decreased the ATP content in a dose-dependent manner at 37°C, it hardly did so at 4°C. Results of studies show that the toxin oligomerizes into a prepore at 4°C and forms a pore at 37°C.

*S*. *aureus* alpha-toxin enters a characteristic cell line such as human keratinocyte cell line HaCaT and African green monkey kidney fibroblast cell line Cos7 cells [20]. Internalization of the toxin plays a role in cellular survival [20]. Therefore, we studied the internalization of delta-toxin in A549 cells. After incubation of A549 cells with Cy3-labeled delta-toxin at 4°C for 30 min, binding to the cell membranes was observed using confocal microscopy (Fig 2D, panel a). Upon transfer of cells from 4°C to 37°C, the persistence of delta-toxin on the cell membrane of A549 cells was detected (Fig 2D, panel b). Results of studies demonstrate that delta-toxin forms a pore in sensitive cell membranes, and that the toxin does not enter the cells.

### Action of delta-toxin on phospholipid-cholesterol liposomes

**Fig 3:**
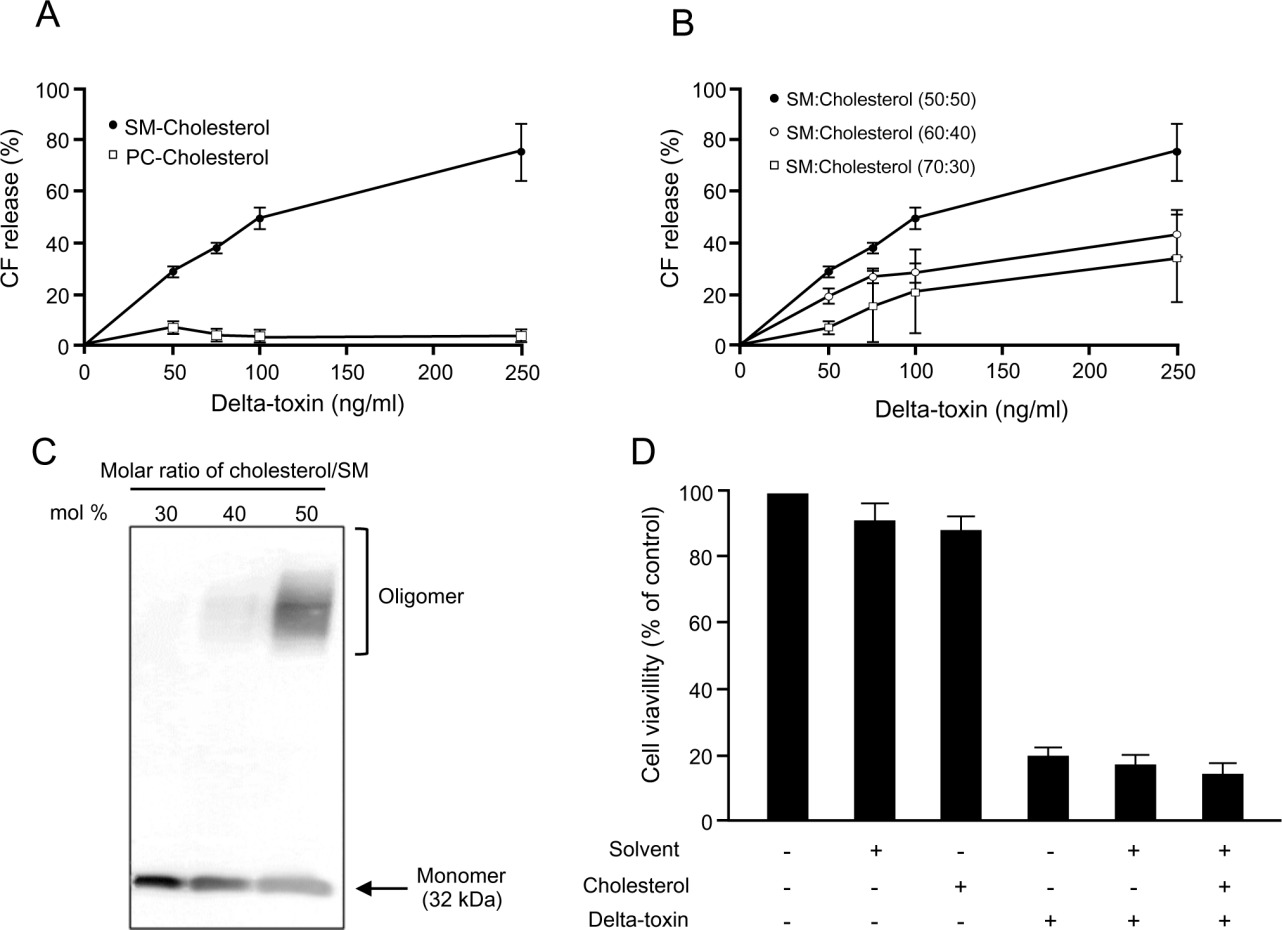
Delta-toxin induced carboxyfluorescein release from phospholipid-cholesterol liposomes. (A) Carboxyfluorescein (CF)-loaded liposomes each composed of SM or PC and cholesterol at a molar ratio of 50:50 mol % were treated with delta-toxin for 30 min at 37°C. (B) Liposomes composed of sphingomyelin (SM) and cholesterol at several molar ratios were treated with delta-toxin for 30 min at 37°C. The molar ratio of cholesterol to SM (mol %) was 50, 40, or 30. CF release was measured as described in the Materials and Methods. The mean ± standard deviation (SD) of four experimental studies is shown. (C) Liposomes composed of SM and cholesterol at various molar ratios (50, 40 or 30 mol %) were treated with delta-toxin (1 μg/ml) for 30 min at 37°C. Liposome-bound toxin was solubilized and confirmed by immunoblotting of delta-toxin. The result is representative of four experimental studies. (D) Effect of cholesterol on cytotoxicity caused by delta-toxin. To assay cholesterol inhibition, a 50 μl volume of cholesterol in absolute ethanol was added to 1 ml aliquots of 50 ng/ml delta-toxin preparations, to a final concentration of 1 μg/ml. After 30 min treatment at room temperature, cytotoxicity was assayed as described in the Materials and Methods. Cell viability was assessed using the 3-(4,5-dimethylthiazol-2-yl)-5-(3-carboxymethoxyphenyl)- 2-(4-sulfophenyl)-2H-tetrazilum (MTS) method. Results are indicated as percentage of the value for controls. The mean ± standard deviation (SD) for four experimental studies is shown.

To test whether delta-toxin forms a pore in artificial lipid membranes, CF-containing liposomes composed of phospholipids and cholesterol at a molar ratio of 50:50 mol % was incubated with various concentrations of delta-toxin for 30 min at 37°C. As shown in Fig 3A, delta-toxin dose-dependently caused CF efflux from bovine brain SM-cholesterol liposomes, but not egg-yolk PC-cholesterol liposomes. To examine the effects of cholesterol on the toxin-induced CF efflux, we prepared liposomes composed of SM containing various proportions of cholesterol. Fig 3B shows that delta-toxin-induced CF-efflux decreased with decreasing amounts of cholesterol. Additionally, the effects of cholesterol content on the binding of toxin to liposomes were examined by SDS-PAGE. After the incubation of SM liposomes containing various cholesterol contents with delta-toxin, the levels of delta-toxin monomer decreased and oligomer increased with increasing cholesterol content in these conditions (Fig 3C). The results of these studies indicate a strong connection between oligomer formation of delta-toxin and toxin-induced CF efflux. On the other hand, the application of cholesterol did not influence the effect on the cytotoxicity induced by delta-toxin (Fig 3D), demonstrating that cholesterol does not directly interact with delta-toxin. These results showed that the toxin binding to liposomes is dependent on SM, and oligomer formation of delta-toxin in liposomes correlates with cholesterol content.

### Association of delta-toxin oligomer with lipid rafts

**Fig 4:**
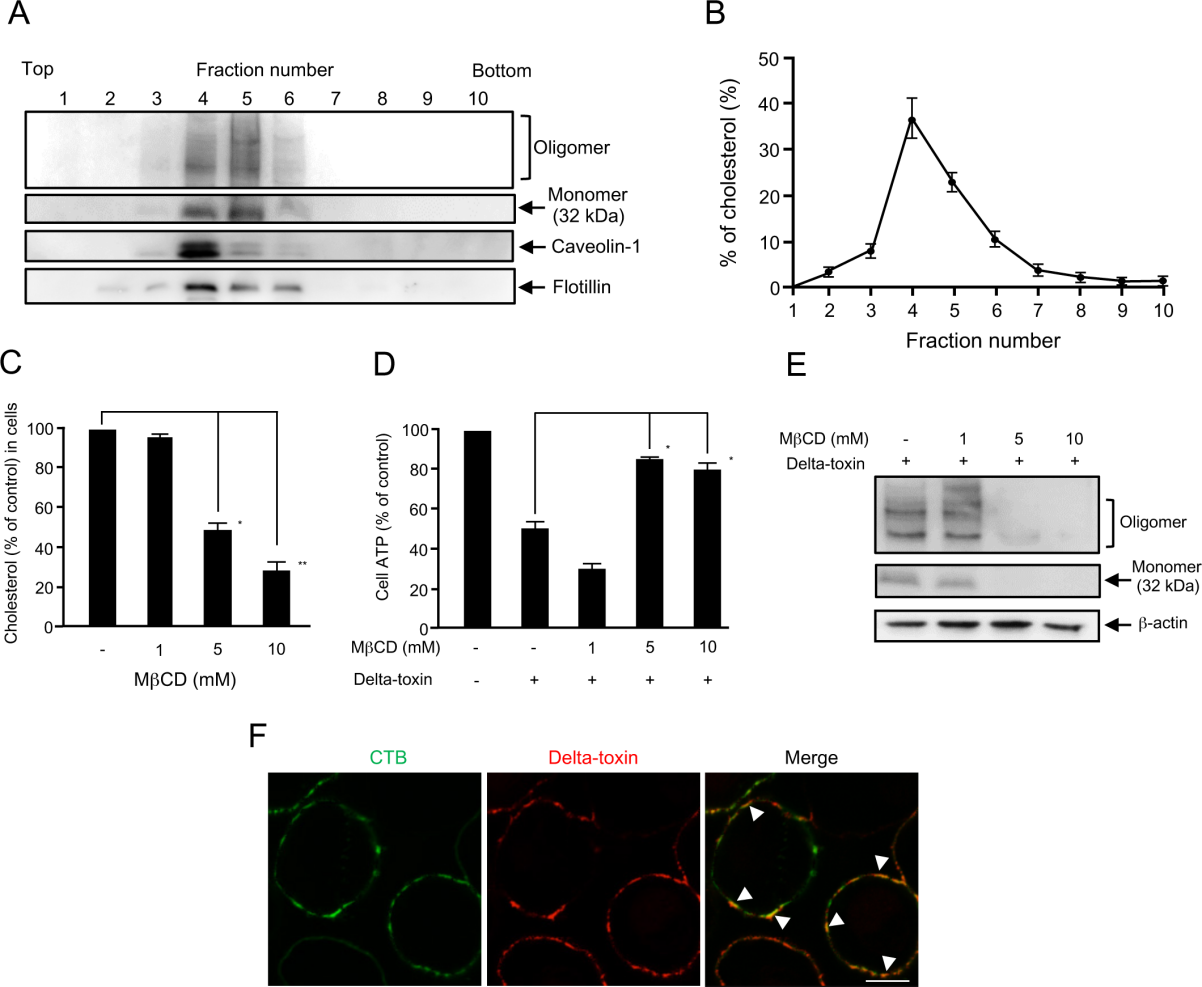
Delta-toxin binding to lipid rafts of A549 cells. (A) A549 cells were treated with delta-toxin (1 μg/ml) for 15 min at 37°C. After washing, the cells were dissolved in 1% Triton X-100. Then, lipid rafts were separated using a sucrose gradient. Portions of the 10 fractions from the gradient were assessed by immunoblotting using anti-delta-toxin, anti-caveolin-1, or anti-flotillin antibodies. The result is representative of four experimental studies. (B) Cholesterol distribution in the fractions of lipid rafts. Cholesterol distribution in lipid raft fractions was accessed as described in the Materials and Methods. (C) A549 cells were incubated with methyl-β-cyclodextrin (MβCD) for 60 min at 37°C. The cholesterol contents were assayed spectrophotometrically as described in the Materials and Methods. (D) A549 cells were treated with MβCD for 60 min at 37°C. The cells were treated with delta-toxin (50 ng/ml) for 30 min at 37°C prior to ATP assays. Data are reported as the percentage of the value for untreated controls. The mean ± standard deviation (SD) of four experimental studies is shown. Results were assessed using one-way ANOVA with Bonferroni’s multiple-comparison post-test. *P<0.05, **P<0.01. (E) A549 cells were incubated with MβCD for 60 min at 37°C. The cells were treated with delta-toxin (1 μg/ml) for 30 min at 37°C. The cells were solubilized in SDS-sample solution and confirmed by immunoblotting using anti-delta-toxin or anti-β-actin antibodies. The result is representative of four experimental studies. (F) Partial colocalization of GM1-rich rafts and delta-toxin. A549 cells were treated with Cy3-labeled delta-toxin (1 μg/ml) and Alexa Fluor 488-labeled cholera toxin B subunit (CTB) (1 μg/ml) for 15 min at 37°C. Delta-toxin (red) and CTB (green) were examined using a confocal microscope. The arrowhead (yellow) shows the colocalization of delta-toxin with CTB. The result is representative of four experimental studies. Bar, 5 μm.

*C*. *perfringens* beta-toxin, a member of the β-PFTs, is shown to oligomerize in the lipid rafts of sensitive cells [16, 21]. To study the potential interaction of delta-toxin with lipid raft fractions of A549 cells, the cells were treated with delta-toxin at 37°C and then incubated with 1% Triton X-100 at 4°C. Membrane components treated with Triton X-100 were separated by flotation centrifugation. Fig 4A shows that the monomer and oligomer forms of delta-toxin were recovered from fractions enriched with lipid raft marker caveolin-1 and flotillin (fractions 3 to 6). Furthermore, >87% of the cholesterol was observed (Fig 4B), which exhibited that these fractions 3 to 6 were lipid rafts. These results show that the monomer and oligomer of delta-toxin are associated with lipid rafts in A549 cells.

It has been shown that methyl-β-cyclodextrin (MβCD) depletes cholesterol and disrupts the integrity of lipid rafts [16]. When A549 cells were treated with 5 and 10 mM MβCD for 60 min at 37°C, the contents of cholesterol reduced by approximately 50% and 32%, respectively, compared with that of control cells (Fig 4C). Incubating A549 cells with 5 and 10 mM MβCD had a protective effect against a quick decrease in cellular ATP induced by delta-toxin (Fig 4D). Moreover, to determine whether delta-toxin associates with MβCD-treated cells, the cells incubated with 1 to 10 mM MβCD were treated with delta-toxin at 37°C. The treated cells were lysed and subjected to immunoblotting analysis of delta-toxin. Fig 4E shows that the intensity of bands of the monomer and oligomer forms of delta-toxin in 5 or 10 mM MβCD-pretreated cells were notably decreased compared with that in control cells. These findings demonstrate that delta-toxin induces cell death in A549 cells through oligomerization in plasma membrane lipid rafts of the sensitive cells.

We investigated whether delta-toxin colocalized with cholera toxin subunit B (CTB), which specifically binds to the raft component GM1 ganglioside [22], on the plasma membrane of A549 cells (Fig 4F). When A549 cells were treated with delta-toxin labeled with Cy3 and Alexa Fluor 488-labeled CTB at 37°C, delta-toxin and CTB bound and partially colocalized on the cell surface, indicating that delta-toxin associated with lipid rafts containing GM1.

### Cell death caused by delta-toxin

**Fig 5:**
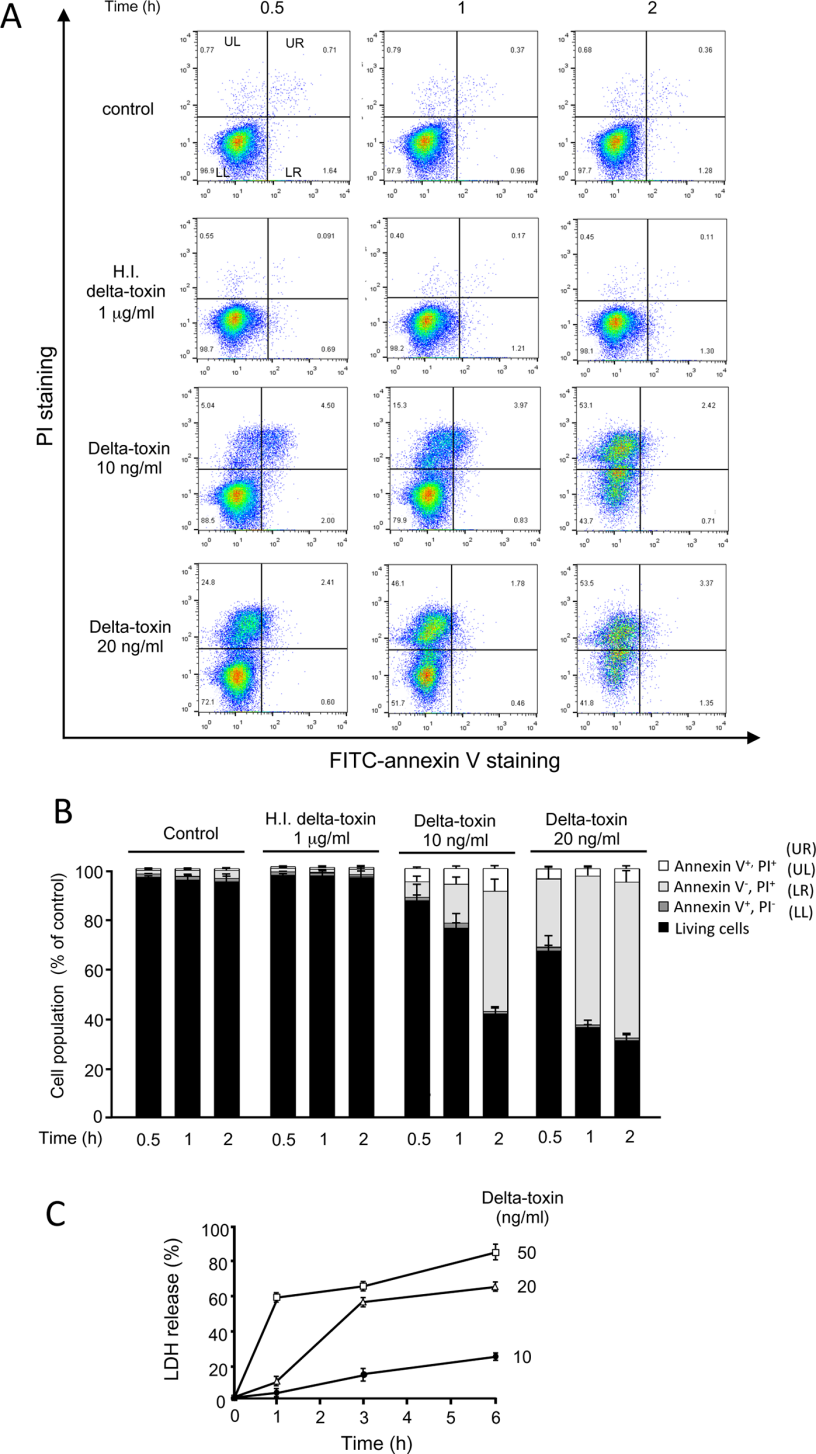
Cytotoxic effect of delta-toxin on A549 cells. (A) A549 cells were treated with heat-inactivated (H.I.) delta-toxin (1 μg/ml) or delta-toxin (10 and 20 ng/ml) for the indicated time periods at 37°C. After washing, the cells were detached using trypsin, stained with propidium iodide (PI) and annexin V, treated for 15 min at 25°C and assessed by flow cytometry. Percentages of cells of each quadrant are shown in each dot-plot graph. Quantitative analysis of the staining is shown in (B). The values are the mean ±s tandard deviation (SD) of four experimental studies in each group. (C) A549 cells were incubated with delta-toxin (10, 20, and 50 ng/ml) at 37°C for the indicated time periods before lactate dehydrogenase (LDH) was measured. The means ± standard deviation (SD) of four independent experiments are shown.

**Fig 6:**
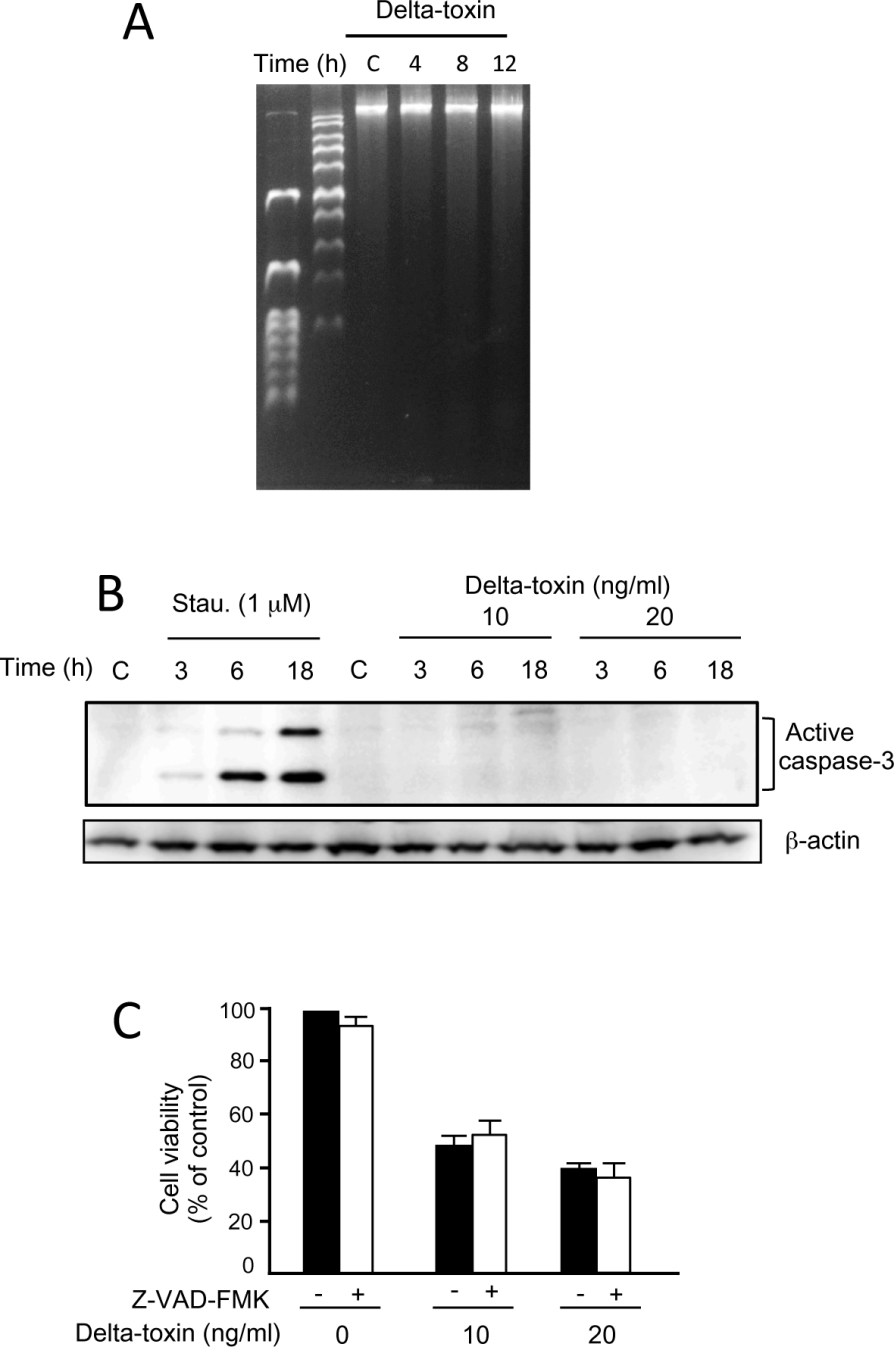
Effect of delta-toxin on caspase signaling in A549 cells. (A) Nuclei from untreated A549 cells (lane C) and those treated with delta-toxin (20 ng/ml) for 4–12 h at 37°C (lanes 4, 8, and 12) was separated by agarose gel electrophoresis. The result is representative of four experimental studies. (B) Delta-toxin did not activate caspase-3 in A549 cells. A549 cells were incubated with staurosporine (Stau.) (1 μM) or delta-toxin (10 or 20 ng/ml) for the indicated time periods at 37°C. After washing, the cells were solubilized with SDS-sample solution and confirmed by immunoblotting analysis using anti-active caspase-3 or anti-β-actin antibodies. The result is representative of four experimental studies. (C) Effect of Z-VAD-FMK on cytotoxicity induced by delta-toxin. A549 cells preincubated with Z-VAD-FMK (25 μM) for 1 h at 37°C were treated with delta-toxin (10 or 20 ng/ml) for 1 h at 37°C. Viability of the cells was assessed using 3-(4,5-dimethylthiazol-2-yl)-5-(3-carboxymethoxyphenyl)-2-(4-sulfophenyl)-2H-tetrazilum (MTS) method. Results are indicated as percentage of the value for controls. The mean ± standard deviation (SD) from four experimental studies is shown.

We studied the possible mechanisms underlying cell death induced by delta-toxin by examining cell death induced by delta-toxin using annexin V and PI staining. Annexin V recognizes the externalization of phosphatidylserine (PS) in the plasma membrane, which is a characteristic property of primary apoptotic cells, whereas PI, a cationic dye, finds necrotic cells with disrupted plasma membranes [23]. A549 cells were incubated with delta-toxin or heat-inactivated delta-toxin for the indicated time periods at 37°C. After incubation, the cells were stained with annexin V/PI and subjected to FCM analysis (Fig 5A and 5B). When cells were incubated without delta-toxin or with heat-inactivated delta-toxin, most of them showed the phenotype of viable cells (annexin V-negative/PI-negative). The number of annexin V-negative and PI-positive cells (UL) started to increase 0.5 h after delta-toxin treatment and continued to increase until 2 h after treatment in a dose-dependent fashion (Fig 5A and 5B). There were almost no increases in the number of annexin V and PI double-positive (UR) cells after 2 h of exposure to the toxin (10 and 20 ng/ml). Necrosis was determined using an LDH release assay. As shown in Fig 5C, delta-toxin induced LDH release from A549 cells in a dose- and time-dependent manner. Moreover, incubation of A549 cells with delta-toxin did not induce DNA ladder formation, a character of apoptosis, after a 12-h incubation period (Fig 6A). No induction of apoptotic caspase-3 was found in delta-toxin-treated cells (Fig 6B). Moreover, the pan-caspase inhibitor Z-VAD-FMK did not inhibit cytotoxicity induced by the toxin, as assessed using MTS assays (Fig 6C). These results indicate that delta-toxin causes cell necrosis.

### Effect of delta-toxin on mitochondria in A549 cells

**Fig 7:**
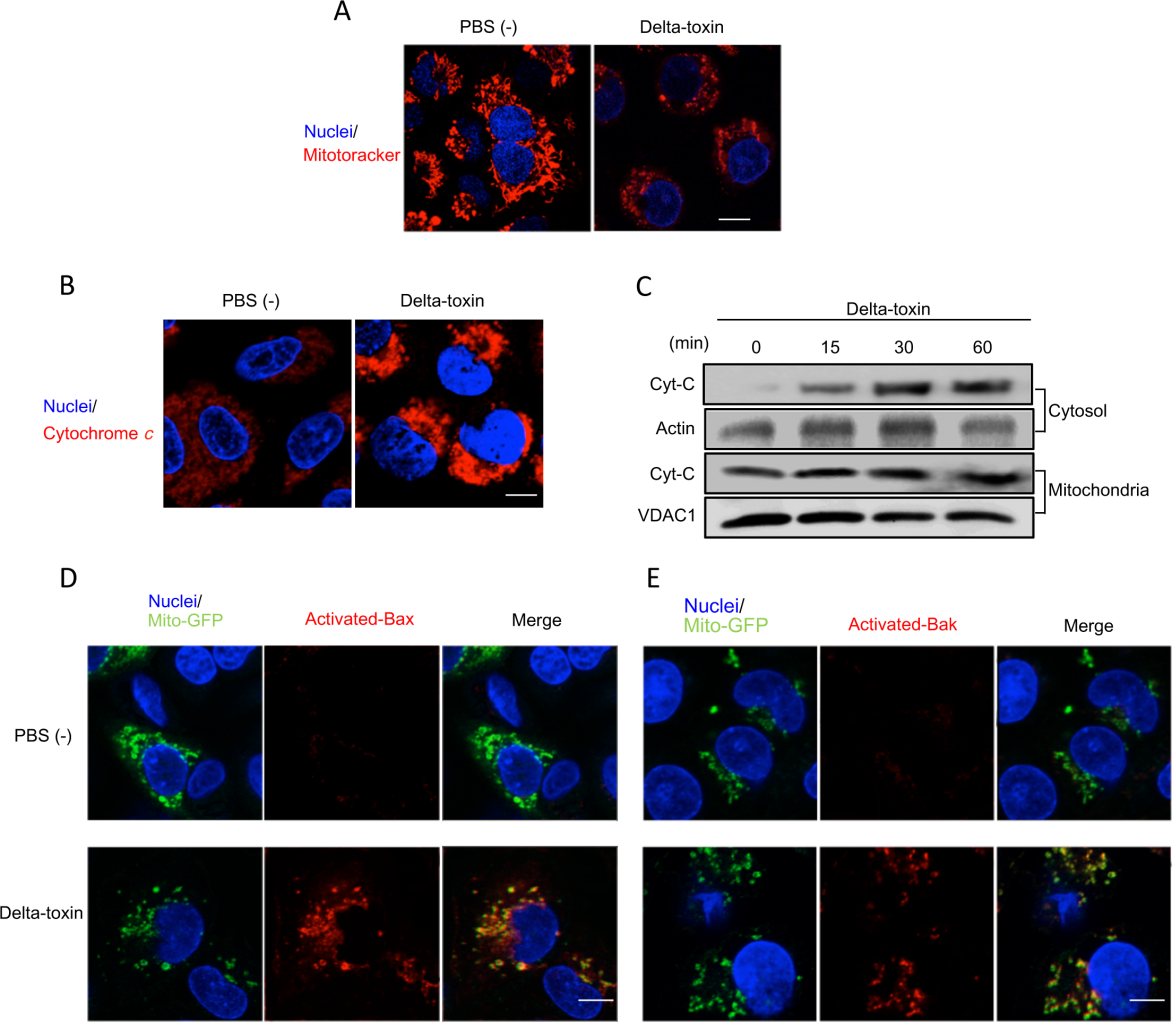
Delta-toxin caused mitochondrial dysfunction. (A) A549 cells preincubated with MitoTracker red and Hoechst 33342 were treated with delta-toxin (50 ng/ml) for 30 min at 37°C. (B) A549 cells were treated with delta-toxin (50 ng/ml) for 30 min at 37°C. Cells were formaldehyde-fixed, permeabilized and stained using an anti-cytochrome *c* antibody and Hoechst 33342. (C) A549 cells were treated with delta-toxin (50 ng/ml) for the indicated time periods at 37°C. The mitochondria and the cytosol fractions were prepared as described in the Materials and Methods, and then were subjected to immunoblotting for the detection of cytochorome *c*. (D,E) A549 cells transfected with Mito-GFP were treated with delta-toxin (50 ng/ml) for 30 min at 37°C. The cells were formaldehyde-fixed, permeabilized and stained with an active-form-specific anti-Bax antibody (D) or an active-form-specific anti-Bak antibody (E). Nuclear DNA was stained with Hoechst 33342. Cells were examined using a confocal microscope. These results are representative of four experimental studies. Bar, 5 μm.

Delta-toxin induced a dramatic decline in the cellular ATP contents of A549 cells within 60 min (Fig 1). Therefore, we examined whether this delta-toxin-induced ATP decrease changed the permeability of mitochondria. Mitochondria were stained with the mitochondrion-specific probe MitoTracker Red, which is incorporated in a mitochondrial membrane potential-dependent manner [17]. As shown in Fig 7A, incorporation of MitoTracker Red into mitochondria in untreated cells occurred uniformly, especially around the nuclei. However, mitochondria in delta-toxin-treated cells showed a dramatic reduction in fluorescence signal and a change in their morphology (Fig 7A). This result indicates that delta-toxin induces the dysfunction of mitochondria in A549 cells. Mitochondrial membrane potential is maintained by cytochrome *c* of the electron transport chain [17], and a reduction in mitochondrial membrane potential induces cytochrome *c* release from mitochondria. We investigated the intracellular distribution of cytochrome *c* in A549 cells treated with delta-toxin. In untreated cells, cytochrome *c* release was detected slightly within mitochondria in their cytoplasm. However, delta-toxin caused cytochrome *c* release from mitochondria to the cytoplasm (Fig 7B). Next, delta-toxin-induced cytochrome *c* release from mitochondria was investigated using cell fractionation (Fig 7C). The results revealed that delta-toxin caused cytochrome *c* release from mitochondria to cytosol. In turn, we employed A549 cells expressing mitochondria-GFP (Mito-GFP) used as a marker for mitochondria. The pro-apoptotic Bcl-2-family protein Bax has been shown to cause mitochondrial permeabilization and cytochrome *c* release. Therefore, we investigated whether cytochrome *c* release from mitochondria induced by delta-toxin was related to Bax activation. The activated form of Bax assembles mainly with the outer membrane of mitochondria [17]. We investigated the intracellular distribution of activated Bax in Mito-GFP expressing A549 cells treated with delta-toxin. The activated form of Bax was detected using an active-form-specific anti-Bax antibody. In control cells, Bax was not revealed in mitochondria (Fig 7D), but the activated form of Bax in delta-toxin-treated A549 cells co-localized with mitochondria. Furthermore, we found the activation of Bak induced by delta-toxin using active-form-specific anti-Bak antibodies (Fig 7E), showing that the activated form of Bak, another Bcl-2 family, co-localized with mitochondria.

## Discussion

Delta-toxin has been demonstrated to induce hemolysis and cause the disruption of various cells [5–8], but the mechanism of cytotoxicity remains unclear. The results presented here are the first to show that delta-toxin induces rapid necrosis via pore formation in the lipid rafts of sensitive cells.

Numerous toxins elaborated by *C*. *perfringens* play a pivotal role in severe infections in humans and animals. Delta-toxin is produced by many *C*. *perfringens* type C strains and also presumably by type B strains [1–3]. The toxin is lethal for mice and cytotoxic to many eukaryotic cells, including rabbit macrophages, human monocytes and platelets derived from humans, guinea pigs, rabbits and goats [6]. Delta-toxin was shown to lyse sensitive cells containing abundant ganglioside GM2 in the membrane [9]. Moreover, it was thought that delta-toxin binds to membrane lipids or proteinaceous toxin receptor [9,12]. In the present study, delta-toxin was found to be cytotoxic for various cell types. The cytotoxicity of the toxin was correlated with its oligomer formation on sensitive cells. Delta-toxin binds and forms oligomers at 4°C and 37°C, but only forms a pore at 37°C in sensitive cells. On the other hand, the oligomers formed in A549 cells incubated with the toxin at 4°C were capable of forming active pores, because the change in those cells to 37°C resulted in quick cell death. Pore formation of a number of PFTs takes place on the surface of the membrane [24], resulting in a prepore step before membrane insertion of the toxin oligomer to form a pore [25–28]. *S*. *aureus* alpha-toxin, also a member of the β-PFT family, oligomerizes into a non-lytic oligomer on the surface of the membrane. This state of the prepore is a characteristic property of β-PFTs [24]. Conformational changes of toxins take place during membrane insertion. *S*. *aureus* alpha-toxin as well as delta-toxin possesses a three-domain structure (β-sandwich, rim, stem) with a predominantly β-structure [29]. To form the characteristic mushroom-shaped oligomer of the active pore, the stem domain protrudes and refolds into a β-hairpin conformation to form a complete transmembrane β-barrel [24, 29]. On the basis of the current study, a proposed model for the cytotoxic effect of delta-toxin is as follows: (I) delta-toxin binds to an unknown receptor; (II) the toxin forms an oligomer into a non-lytic prepore on the surface of the membrane; and (III) the prepore at 37°C inserts into the membrane to form a pore. The present study also indicated a prepore stage in the action of delta-toxin.

Manich *et al*. [9] reported that in an artificial PC bilayer membrane delta-toxin forms slightly anion-selective channels. In this study, delta-toxin caused CF release from SM-cholesterol liposomes but not PC-cholesterol liposomes. On the other hand, cholesterol did not affect the delta-toxin-induced activity. Because delta-toxin did not bind to SM [4], binding of the toxin occurred when both cholesterol and SM were present. As shown here, delta-toxin monomer bound to liposomes composed of SM and cholesterol, and pore formation of the toxin in the lipid bilayer membranes correlated with cholesterol content. The role of the liposome composition in the function of β-PFTs has been reported [18, 30], and it has been reported that cholesterol may allow the clustering of phosphocholine head groups in SM-cholesterol microdomains [14, 30, 31]. The phosphocholine headgroups of SM, clustered in the SM-cholesterol microdomain, acted as a high-affinity receptor for *S*. *aureus* α-toxin [31]. On the other hand, PC could not functionally replace SM in this liposomal system. Cholesterol modulates the membrane fluidity of phospholipid bilayers. Although the exact role of liposome composition in delta-toxin function needs to be investigated further, we propose that delta-toxin binds to the clustered phosphorylcholine in SM-cholesterol liposomes and forms a pore through the increased membrane fluidity contributed to by cholesterol. Possibly, cholesterol plays a role in the proper orientation of phosphocholine head groups for toxin binding.

Delta-toxin monomer and oligomer were detected in lipid rafts. The incubation of A549 cells with MβCD decreased the content of cholesterol in lipid rafts, toxin binding to cells and the cytotoxicity of the toxin. Oligomerization of β-PFT is commonly accelerated in cholesterol-enriched microdomains or lipid raft fractions, which work as concentrating platforms [14, 32]. Valeva *et al*. [31] reported that SM-cholesterol microdomains indeed represented the“specific”binding sites for *S*. *aureus* alpha toxin. From these findings, delta-toxin binds to the SM-cholesterol microdomain in lipid rafts and assembles into a pore through the prepore oligomer.

We showed that delta-toxin caused swelling of the cells and cytotoxicity. Delta-toxin caused the activation of pro-apoptotic Bcl-2 family members Bak and Bax, already known to induce mitochondrial permeability, and cytochrome *c* release into the cytosol [33, 34]. On the other hand, cleaved caspase-3 was not detectable in the cytosol from delta-toxin-treated cells. Furthermore, pretreating A549 cells with the pan-caspase inhibitor Z-VAD-FMK did not inhibit delta-toxin-induced cytotoxicity. Additionally, delta-toxin did not lead to apoptotic DNA ladder formation. Results of studies show that although delta-toxin caused the activation of Bax and Bak, the mechanism of cytotoxicity induced by delta-toxin was not attributed to the activation of a caspase-mediated apoptosis. On the other hand, annexin V-negative and PI-positive cell populations increased after delta-toxin treatment. Furthermore, the toxin caused marked ATP depletion, which is one of the initial stages of necrosis. Delta-toxin-mediated increases in annexin V-negative and PI-positive cell populations were associated with the loss of cell survival, furthering our understanding of the view that delta-toxin induced necrosis. Cellular ATP concentrations reduced to about 93% of controls during 60 min of delta-toxin application, indicating that a large fraction of cells were assumed to be necrotic, because the cells lacked adequate ATP to support energy-mediated apoptosis. Delta-toxin exposure leads to mitochondrial membrane permeabilization, as shown by the release of cytochrome *c* into the cytosol and the reduced staining of MitoTracker. Mitochondrial damage played an important role in the cytotoxic effect; in fact, serious depletion of ATP could be enough to induce cell death. The data demonstrate that delta-toxin causes damage to mitochondria, which in turn seems to lead to depletion of ATP. The pro-apoptotic Bcl-2 family members Bak and Bax are both definitive regulatory factors of mitochondrial permeabilization [34]. In response to the induction of apoptosis, Bax protein changes conformation, exposing membrane-targeting domains and resulting in its translocation of mitochondrial membrane [33]. Subsequently, Bax inserts and induces cytochrome *c* release [33]. Bak is mostly coupled with the outer membrane of mitochondria in non-apoptotic cells. It also alters conformation through an apoptotic signal. Activated Bax and Bak give rise to homo-oligomerization on mitochondria and are involved in the formation of a mitochondrial permeability transition pore that accelerates cytochrome *c* release. Here, we indicated that Bak and Bax were activated and localized to mitochondria in A549 cells exposed to delta-toxin. The present study shows that delta-toxin may employ both of Bcl-2 proteins to cause mitochondrial damage. The present study discovered that delta-toxin was bound to the plasma membrane in A549 cells in which the release of cytochrome *c* had been caused. The findings demonstrated that a direct interaction between delta-toxin and mitochondria is unnecessary for delta-toxin-induced cytochrome *c* release. Namely, another pathway involved in Bak and Bax activation seems to be responsible for the actions of delta-toxin.

Delta-toxin was elaborated by *C*. *perfringens* type C strains that cause severe infection in animals [2, 3]. However, the role of delta-toxin in the virulence of intestinal infections remains unknown. In this study, we showed that delta-toxin caused rapid cell necrosis in various cells. On the basis of these findings, it is possible that delta-toxin-induced cell injury is related to the establishment of infection by *C*. *perfringens* type C.

To summarize, we indicated that delta-toxin induces the quick ATP depletion and necrosis in sensitive cells. Therefore, the cells provide an effective approach that is required to solve the cytotoxic mechanism of delta-toxin.
